# Why should one normalize heart rate variability with respect to average heart rate

**DOI:** 10.3389/fphys.2013.00306

**Published:** 2013-10-22

**Authors:** Jerzy Sacha

**Affiliations:** Department of Cardiology, Regional Medical CenterOpole, Poland

**Keywords:** heart rate variability, heart rate, autonomic nervous system, analysis, R-R interval

Heart rate variability (HRV) is a recognized risk factor in many disease states (Bravi et al., 2011; Sacha et al., 2013a). However, HRV is significantly correlated with an average heart rate (HR), and this association is both physiologically and mathematically determined. The physiological determination comes from the autonomic nervous system activity (Task Force of the European Society of Cardiology, and the North American Society of Pacing and Electrophysiology, 1996), but the mathematical one is caused by the non-linear (inverse) relationship between R-R interval and HR (Sacha and Pluta, 2005a,b, 2008).

**Figure 1:**
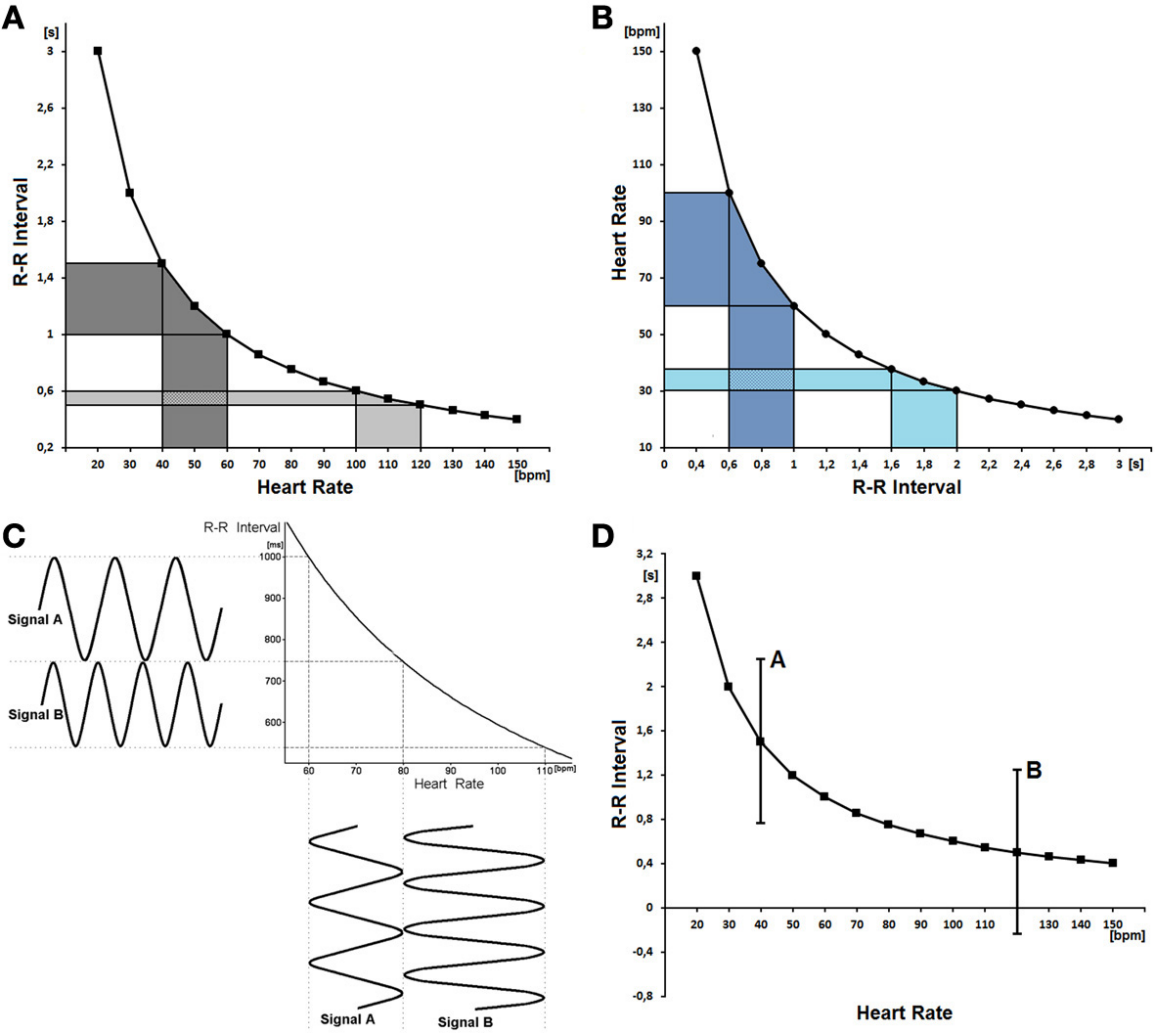
**(A)**The non-linear (mathematical) relationship between R-R interval and heart rate is depicted. One can see that the oscillations of a slow average heart rate (*x*-axis, dark gray area) result in much greater oscillations of RR intervals (*y*-axis, dark gray area) than the same oscillations of a fast average heart rate (light gray area). As a consequence, the variability of R-R intervals is higher for the slow average heart rate than for the fast one, despite the fact that the variability of heart rate is the same (reprinted with modification from Sacha and Pluta, 2008). **(B)** The relationship between heart rate and R-R interval is shown—the same oscillations of R-R intervals yield much greater oscillations of HR for the fast average heart rate (dark blue area) than for the slow one (light blue area). Consequently, the variability of HR is higher for the case with fast average heart rate, despite the fact that the variability of R-R intervals is the same in both cases. **(C)** The relationship between R-R interval and heart rate is depicted along with two signals oscillating in different extents. Signal A oscillates between 60 and 80 bpm but signal B between 80 and 110 bpm. One can see that signal A is more variable (its amplitude is higher) than signal B when expressed as R-R interval signals, and conversely signal A is less variable than B if expressed as HR ones. The example clearly shows how the same signals may reveal an inverse relationship with each other depending on the way they are expressed (reprinted from Sacha and Pluta, 2005a). **(D)** The relationship between R-R interval and heart rate with two hypothetical examples of R-R interval oscillations (i.e., A and B) are presented. It is shown that the fluctuations of R-R intervals may be potentially quite high for a slow average HR (A), however, such fluctuations are not possible for a fast average HR (B) since the R-R intervals should have become negative.

HRV may be estimated by using R-R interval (the most frequent method) or HR signals, yet, they both do not yield the same results since they are inversely related with each other—indeed, the analyses are mathematically biased (Sacha and Pluta, 2005a,b). If one uses R-R intervals, the same changes of HR cause much higher fluctuations of R-R intervals for the slow average HR than for the fast one (Figure 1A). Conversely, if one employs HR signals, the same changes of R-R intervals cause much higher fluctuations of HR for the fast than slow average HR (Figure 1B). Consequently, due to these mathematical reasons, HRV estimated from R-R intervals should negatively correlate with average HR (or positively with average R-R interval), but HRV estimated from HR signals should be positively correlated with average HR (or negatively with average R-R interval) (Sacha and Pluta, 2005a,b). Moreover, due to the inverse relationship between R-R interval and HR, there is a possibility that a given patient may have higher HRV than another in terms of R-R intervals and lower HRV in terms of HRs—Figure 1C explains such a case (Sacha and Pluta, 2005a).

Another mathematical problem concerning the association between HRV and HR is presented in Figure 1D. One can see that the fluctuations of R-R intervals may be potentially very high for slow average HR, however, the same fluctuations are not possible for fast average HR, since the R-R intervals should have become negative. The same problem can be met if one calculates HRV from HR signals, i.e., the average HR of 80 bpm may potentially fluctuate between 30 and 130 bpm (i.e., the fluctuation amplitude equals 100 bpm), however, such fluctuations are not possible for the average HR of 40 bpm, since the heart rhythm must have fluctuated between −10 and 90 bpm.

Due to the above facts, the standard HRV analysis is mathematically biased, particularly if patients differ in terms of their average HRs. The only way to overcome it is to calculate HRV with respect to the average value, i.e., to normalize the fluctuations with respect to the mean (Sacha and Pluta, 2005a,b, 2008). One can do that by division of the signal by the average R-R interval in the case of R-R interval signal, or by the average HR in the case of HR signal. Moreover, this way the same results are obtained no matter if one calculates HRV from R-R intervals or HRs (Sacha and Pluta, 2005a).

Such an approach enables to explore the HR contribution to the physiological and clinical significance of HRV (Billman, 2013; Sacha et al., 2013a). Recently, this approach has been further developed to enhance or completely remove the HR influence (even physiological one) on HRV, what turned out to provide valuable information on cardiac and non-cardiac prognosis in patients after myocardial infarction—the details have been published elsewhere (Sacha et al., 2013a,b,c).

To conclude, HRV is significantly associated with HR, which is caused by both physiological and mathematical phenomena, however, by a simple mathematical modification one may exclude mathematical bias and explore a real clinical value of HR and its variability.
